# High Nitrogen Enhance Drought Tolerance in Cotton through Antioxidant Enzymatic Activities, Nitrogen Metabolism and Osmotic Adjustment

**DOI:** 10.3390/plants9020178

**Published:** 2020-02-01

**Authors:** Asif Iqbal, Qiang Dong, Xiangru Wang, Huiping Gui, Hengheng Zhang, Xiling Zhang, Meizhen Song

**Affiliations:** State Key Laboratory of Cotton Biology, Institute of Cotton Research of Chinese Academy of Agricultural Sciences, Anyang 455000, China; asif173aup@gmail.com (A.I.); dongqiang@caas.cn (Q.D.); wxr_z4317@163.com (X.W.); huiping.828@163.com (H.G.); zhanghengheng1314@163.com (H.Z.)

**Keywords:** cotton, nitrogen, drought stress, nitrogen metabolism, enzyme activities, osmotic adjustment

## Abstract

Drought is one of the most important abiotic stresses and hampers many plant physiological processes under suboptimal nitrogen (N) concentration. Seedling tolerance to drought stress is very important for optimum growth and development, however, the enhancement of plant stress tolerance through N application in cotton is not fully understood. Therefore, this study investigates the role of high N concentration in enhancing drought stress tolerance in cotton. A hydroponic experiment supplying low (0.25 mM) and high (5 mM) N concentrations, followed by 150 g L^−1^ polyethylene glycol (PEG)-induced stress was conducted in a growth chamber. PEG-induced drought stress inhibited seedling growth, led to oxidative stress from excessive malondialdehyde (MDA) generation, and reduced N metabolism. High N concentrations alleviated oxidative damage and stomatal limitation by increasing antioxidant enzymatic activities, leaf relative water content, and photosynthesis in cotton seedlings under drought stress. The results revealed that the ameliorative effects of high N concentration may be ascribed to the enhancement of N metabolizing enzymes and an increase in the amounts of osmoprotectants like free amino acids and total soluble protein. The present data suggest that relatively high N concentrations may contribute to drought stress tolerance in cotton through N metabolism, antioxidant capacity, and osmotic adjustment.

## 1. Introduction

Drought is one of the most important abiotic stresses and is associated with the hydrology and climate of an area [1]. China is considered a drought-prone country, due to variations in its monthly and annual rainfall and temperature [2], and drought is a natural phenomenon in almost all regions [3]. Drought stress adversely affects many plant physiological processes [4] such as photosynthesis and carbon and nitrogen metabolism [5]. The sensitivity of crops to drought stress is reflected by a reduction in chlorophyll pigments and photosynthesis, and changes in gene expression and enzymatic activities, which lead to poor growth and production [6,7]. The limitations to photosynthesis induced by drought are attributed to either stomatal or non-stomatal limitations [8]. The drought induced non-stomatal limitations include decreases in linear electron transport, maximum quantum yield, and actual quantum yield of electron flow through photosystem II [9]. These decreases are, in part, associated with the downregulation of the light reactions through processes such as increased non-photochemical quenching [10]. As a result of decreased ATP and ATP synthase levels, drought can limit substrate regeneration for carbon reactions [11]. Still, compelling arguments for a predominantly diffusional limitation under mild or moderate drought are more common [12,13]. However, drought typically has little effect on primary photochemistry for field-grown cotton due to energy dissipation through photorespiration [13,14]. Moreover, the physiological responses induced by drought may help to reduce water loss due to high external osmotic pressure, protect the subcellular structure, and stabilize plant metabolic processes [15]. In addition, compatible solutes like sugars and amino acids (particularly proline) are produced and accumulated in cotton during drought stress, which facilitates osmotic adjustment [15]. The accumulation of these solutes enhances the functional capabilities of stressed plants, but this varies among plant species, cultivars, and plant parts [16]. At present, the mechanism of drought stress tolerance and osmotic adjustment to maintain metabolic function in cotton remains unclear.

Like water, nitrogen is very important for plant growth and productivity [17]. Generally, drought stress affects nitrogen uptake and transport to the aboveground plant parts because it decreases the transpiration rate and membrane permeability. Another reason for the poor transport and metabolism of nitrogen is that both nitrate and ammonium need water for uptake and translocation to the aboveground plant parts for metabolism [17]. The regulation of nitrogen metabolism is very important for plant stress tolerance as most plant physiological processes are associated with it [18]. A previous report stated that the reduction in photosynthesis under drought stress was due to lowered nitrogen metabolism [19]. Many enzymes are involved in nitrogen metabolism, among which glutamine synthetase (GS; EC 6.3.1.2) and glutamate synthase (GOGAT) are very important for the production of amino acids and amides from inorganic nitrogen (N) [17]. Drought has been reported to restrict plant growth through enzymes inhibition implicated in N metabolism as well [15,20,21]. For instance, Pawar et al. [20] demonstrated that drought stress leads to decreased nitrate reductase (NR), GS, and GOGAT enzyme activities. Inhibition of these enzymes increases the concentration of ammonium, which is toxic for plants; therefore, an increase in the ability of a plant to assimilate ammonium can improve stress tolerance [22]. Thus, N metabolism is very important for stress alleviation; however, whether high N concentration can alleviate the negative impact of drought stress in cotton is still unknown.

Previously, many scientists reported that nitrogen can enhance drought tolerance in crops such as rice [23], *Abies fabri* [24], *Picea asperata* [25], and *Pinus pinea* [26]. However, the function of nitrogen as a stress regulator or in stress tolerance depends on the stress intensity and level of nitrogen application [27]. Sufficient nitrogen supports better plant growth and stress tolerance [28]. Previous studies suggested that application of relatively high N concentration results in better growth [29] and nitrogen metabolism, which enhances plant tolerance against stress [27]. Similarly, a high supply of nitrogen increases the plasticity of root development under drought stress in rice [30]. High N concentration also improves photosynthetic traits, which help to mitigate drought stress by increasing the sensitivity of stomatal conductance and maintaining a higher photosynthetic rate in rice [31]. The increased photosynthetic resistance against drought stress under high N concentration is also possibly due to an increase in enzymatic and non-enzymatic antioxidant activities [28]. Moreover, under high N concentration, more N is used to form soluble nitrogenous compounds such as free amino acids like proline, which act as osmotica to balance the water status in stress conditions [32]. Thus, to ensure high productivity in cotton, we need to better understand N metabolism and osmotic regulation through antioxidant enzymatic activities and osmoprotectants in response to drought stress under high N concentration.

Cotton is a widely adapted crop that is grown worldwide in different regions and experiences periodic drought and rewetting cycles. The enhancement of plant stress tolerance due to high N application is already known in other crops; however, the specific underlying mechanisms are still not fully understood in cotton. Therefore, enhancing the potential of cotton to tolerate drought stress will become a scientific and economic issue in the coming decades. To understand the role of high N concentration in drought stress alleviation in cotton, we examined various morphological, physiological, and biochemical changes associated with N metabolism in response to low and high N concentration under polyethylene glycol (PEG)-induced drought stress. We hypothesized that high N concentration might contribute to the enhancement in drought stress tolerance by increasing N metabolism, accumulation of osmotica, and antioxidant enzymatic activities in cotton.

## 2. Results

**Figure 1:**
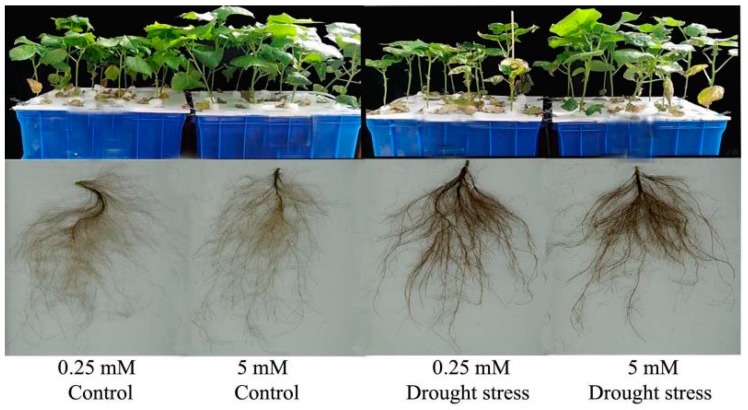
Shoot and root phenotypes of cotton seedlings treated with low and high nitrogen (N) concentration under control and drought stress conditions.

In the current study, drought stress had an adverse effect on the shoot and root phenotypes of cotton seedlings (Figure 1). Therefore, various morphological, physiological, and biochemical traits were measured to assess the adverse impact of drought stress on cotton seedlings and its alleviation through high N concentration.

### 2.1. Plant Morphology and Leaf Relative Water Content

**Figure 2:**
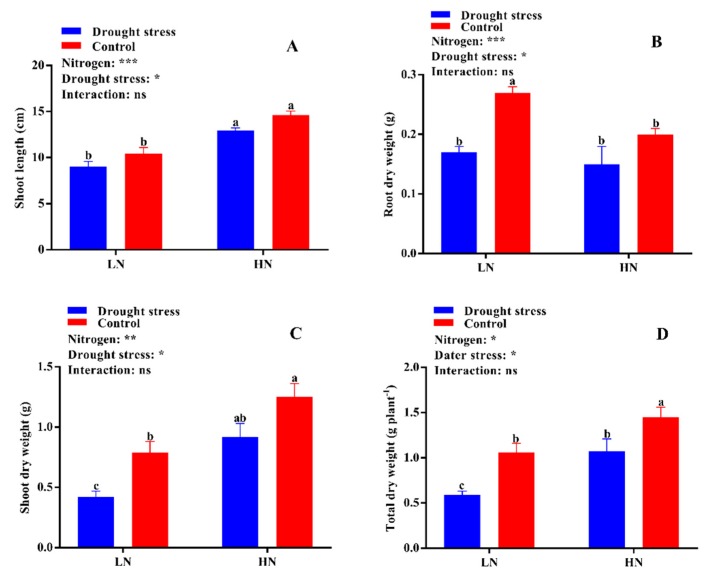
(**A**) Shoot length (cm), (**B**) root dry weight (g), (**C**) shoot dry weight (g), (**D**) total dry weight (g plant^−1^) of cotton seedlings at the end of the experiment. Control: 0 g L^−1^ polyethylene glycol (PEG)-6000; drought stress: 150 g L^−1^ PEG-6000; LN, low N concentration (0.25 mM); HN, high N concentration (5 mM). Bars with different letters indicate significant difference (*p* < 0.05). Error bars represent the standard error (*n* = 3). *p*-Values of the ANOVA of nitrogen, drought, and their interaction are indicated as ns, not significant; * *p* < 0.05, ** *p* < 0.01, *** *p* < 0.001.

**Figure 3:**
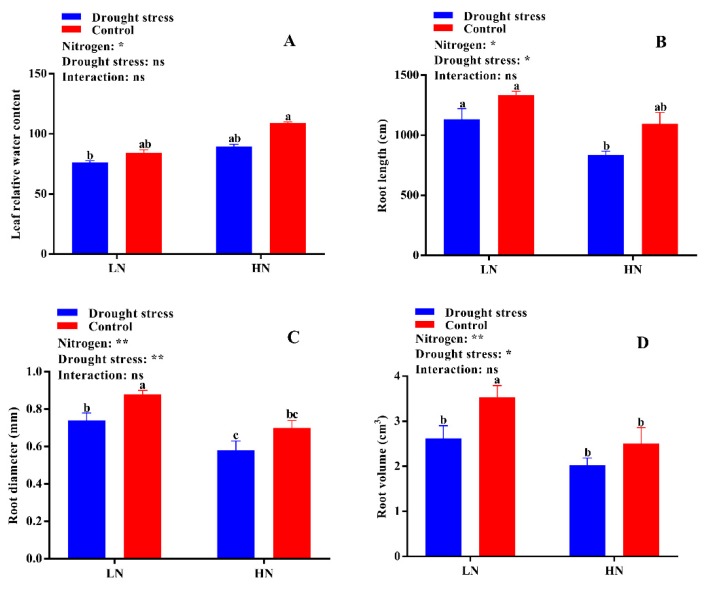
(**A**) Leaf relative water content (%), (**B**) root length (cm), (**C**) root diameter (mm), and (**D**) root volume (cm^3^) of cotton seedlings at the end of the experiment. Control: 0 g L^−1^ PEG-6000; drought stress: 150 g L^−1^ PEG-6000; LN, low N concentration (0.25 mM); HN, high N concentration (5 mM). Bars with different letters indicate significant difference (*p* < 0.05). Error bars represent the standard error (*n* = 3). *p*-Values of the ANOVA of nitrogen, drought, and their interaction are indicated as ns, not significant; * *p* < 0.05, ** *p* < 0.01.

The growth and dry biomass of cotton plants supplied with nitrogen were greatly affected by drought stress, which led to a significant reduction in shoot length (29%) and shoot (44%) and total plant dry weight (35%), while root dry weight increased by 27% under low supply concentration. Drought stress also reduced shoot length and root, shoot, and total plant dry biomass by 12%, 31%, 34%, and 34%, respectively (Figure 2). In contrast, plants grown under high N concentration had lower root dry biomass; however, shoot length and shoot and total plant dry biomass were higher than under low N concentration. In drought stress conditions, plants treated with high N concentration tended to maintain comparatively higher shoot length and dry biomass than those under low N concentration (Figure 2). A relatively higher root:shoot ratio was observed under low N concentration, while the single leaf area and leaf relative water content were higher under high Nconcentration (Figure S1 and Figure 3A). Thus, an increase in leaf relative water content and single leaf area under drought stress meant that high N concentration decreased the impact of drought stress on cotton seedlings. Moreover, the higher root:shoot ratio in cotton seedlings under low N concentration indicated that low N concentration reduced aboveground growth and increased belowground growth (Figure S1).

Root morphology under drought stress was compared for cotton seedlings grown under low and high N concentrations in a hydroponic culture (Figure 1). Drought stress greatly inhibited root growth as indicated by shorter root length (19%) and lower surface area (23%), diameter (17%), and volume (23%) compared with the control under both N concentrations. The difference in root length and surface area was not significant; however, root diameter and volume were significantly lower under high N concentration. Moreover, low N concentration increased root diameter (27%) and volume (36%), which indicated that under drought stress the roots had a great capacity to increase their diameter and volume when the supply of N was low (Figure 3). The root length ratio was significantly different between control and drought stress conditions under low nitrogen concentration, but the root mass ratio was significantly increased by low N concentration under both drought stress and control conditions. In contrast, both the root length ratio and root mass ratio were suppressed under high N concentration. However, the differences between root thickness and root density were not significant under either different drought stress or N concentrations. Unlike shoot morphological traits, root traits were enhanced by low N concentration rather than high N concentration, indicating that under both drought and low N concentration the plant translocated most of the dry matter to improve the root system for better N uptake (Figure S2).

### 2.2. Leaf Physiological Traits

**Figure 4:**
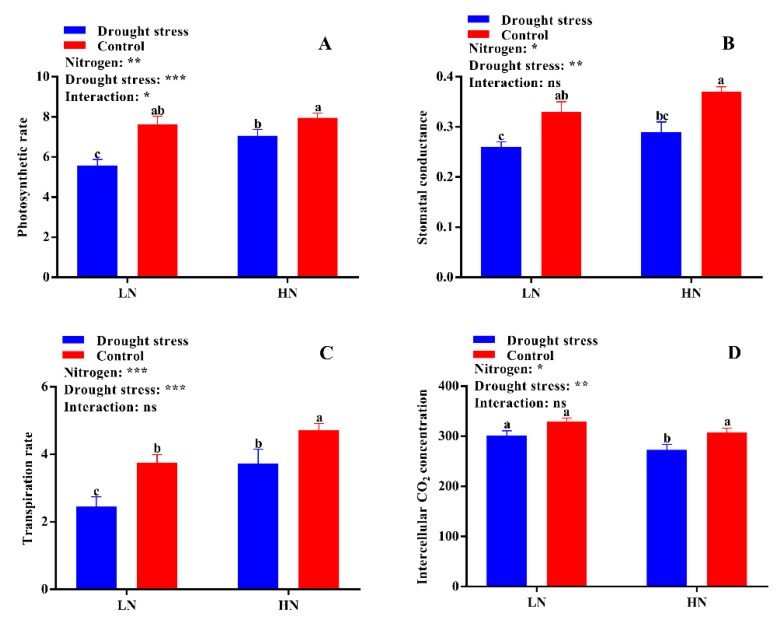
(**A**) Photosynthetic rate (µmol m^−2^ s^−1^), (**B**) stomatal conductance (mmol H_2_O m^−2^ s^−1^), (**C**) transpiration rate (mmol m^−2^ s^−1^), and (**D**) intercellular CO_2_ concentration (µmol CO_2_ mol^−1^ air) of cotton seedlings at the end of the experiment. Control: 0 g L^−1^ PEG-6000; drought stress: 150 g L^−1^ PEG-6000; LN, low N concentration (0.25 mM); HN, high N concentration (5 mM). Bars with different letters indicate significant difference (*p* < 0.05). Error bars represent the standard error (*n* = 3). *p*-Values of the ANOVA of nitrogen, drought, and their interaction are indicated as ns, not significant; * *p* < 0.05, ** *p* < 0.01, *** *p* < 0.001.

Leaf physiological traits like photosynthesis, stomatal conductance, transpiration rate, intercellular CO_2_ concentration, chlorophyll, and carotenoids contents were significantly altered in drought stress-treated plants under low and high N concentrations. The results showed that drought stress significantly suppressed the photosynthetic rate (19%), stomatal conductance (22%), and transportation rate (27%), while the intercellular CO_2_ (10%) concentration was higher as compared to the control condition (Figure 4). However, high N concentration significantly increased photosynthesis (12%), stomatal conductance (12%), and the transpiration rate (26%) compared to low N concentration in both drought stress and control conditions (Figure 4). These results indicated that high N concentration can alleviate the negative impact of drought by increasing dry matter production through leaf photosynthesis.

**Figure 5:**
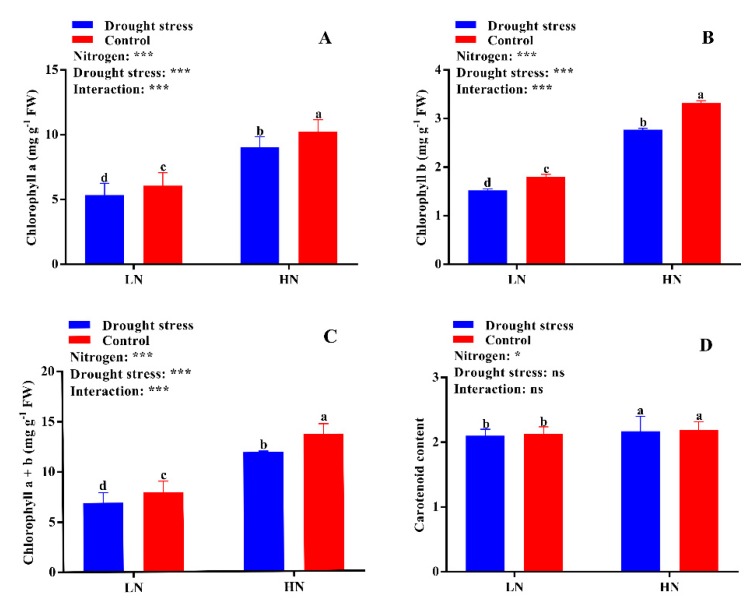
(**A**) Chlorophyll *a* (mg g^−1^ FW), (**B**) chlorophyll *b* (mg g^−1^ FW), (**C**) chlorophyll *a* + *b* (mg g^−1^ FW), and (**D**) carotenoid (g) contents of cotton seedlings at the end of the experiment. Control: 0 g L^−1^ PEG-6000; drought stress: 150 g L^−1^ PEG-6000; LN, low N concentration (0.25 mM); HN, high N concentration (5 mM). Bars with different letters indicate significant difference (*p* < 0.05). Error bars represent the standard error (*n* = 3). *p*-Values of the ANOVA of nitrogen, drought, and their interaction are indicated as ns, not significant; * *p* < 0.05, *** *p* < 0.001.

Drought stress reduced the contents of chlorophyll *a* (12%), chlorophyll *b* (16%), and chlorophyll *a* + *b* (13%) as compared to the control condition (Figure 5). The SPAD value was also significantly decreased by drought stress under both low and high N concentrations as compared to the control (Figure S1). However, cotton seedlings supplemented with high N concentration significantly increased chlorophyll *a* (41%), chlorophyll *b* (46%), chlorophyll *a* + *b* (42%), and carotenoid (3%) contents as compared to low N concentration under both drought and control conditions. These results suggest that high N concentration can alleviate the adverse effects of drought stress on chlorophyll and carotenoids contents and as a result increase the photosynthetic activity of cotton seedlings (Figure 5).

### 2.3. Malondialdehyde (MDA) Content and Superoxide Dismutase (SOD), Peroxidase (POD) and Catalase (CAT) Activities

**Figure 6:**
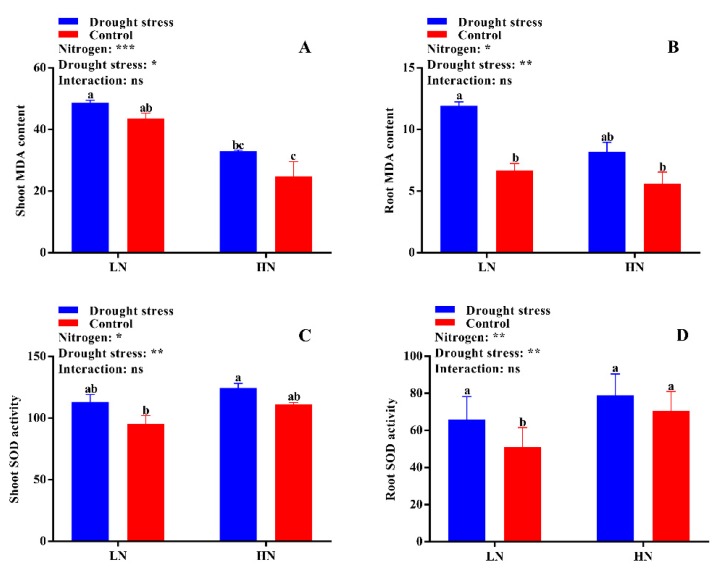
(**A**) Shoot MDA content (mmol g^−1^ FW), (**B**) root MDA content (mmol g^−1^ FW), (**C**) shoot SOD activity (U g^−1^ FW), and (**D**) root SOD activity (U g^−1^ FW) of cotton seedlings at the end of the experiment. Control: 0 g L^−1^ PEG-6000; drought stress: 150 g L^−1^ PEG-6000; LN, low N concentration (0.25 mM); HN, high N concentration (5 mM). Bars with different letters indicate significant difference (*p* < 0.05). Error bars represent the standard error (*n* = 3). *p*-Values of the ANOVA of nitrogen, drought, and their interaction are indicated as ns, not significant; * *p* < 0.05, ** *p* < 0.01, *** *p* < 0.001.

**Figure 7:**
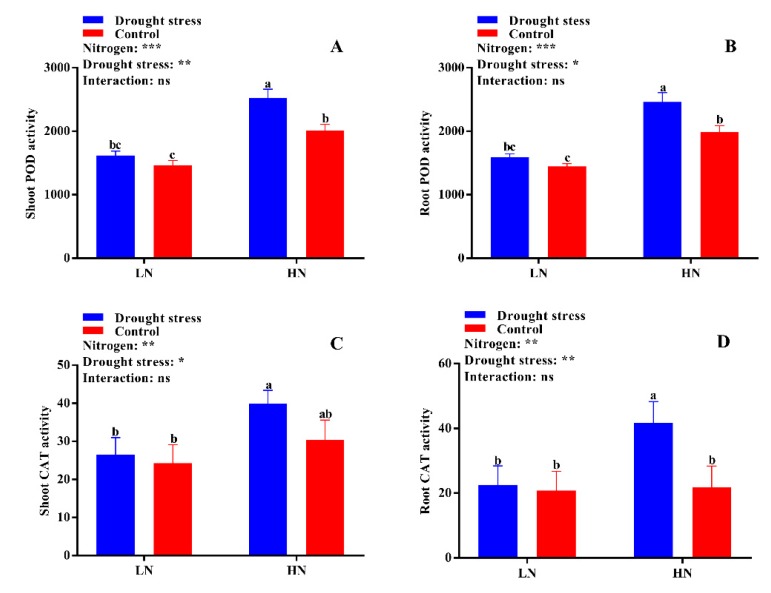
(**A**) Shoot POD activity (U g^−1^ min^−1^ FW), (**B**) root POD activity (U g^−1^ min^−1^ FW), (**C**) shoot CAT activity (U g^−1^ min^−1^ FW), and (**D**) root CAT activity (U g^−1^ min^−1^ FW) of cotton seedlings at the end of the experiment. Control: 0 g L^−1^ PEG-6000; drought stress: 150 g L^−1^ PEG-6000; LN, low N concentration (0.25 mM); HN, high N concentration (5 mM). Bars with different letters indicate significant difference (*p* < 0.05). Error bars represent the standard error (*n* = 3). *p*-Values of the ANOVA of nitrogen, drought, and their interaction are indicated as ns, not significant; * *p* < 0.05, ** *p* < 0.01, *** *p* < 0.001.

No significant difference was found in shoot malondialdehyde (MDA) content between the drought stress and control treatments (Figure 6A). However, the shoot MDA content was 59% higher under low N concentration compared with high N concentration (Figure 6B). Under high N concentration, no significant difference was observed between drought and control conditions for both shoot and root MDA contents (Figure 6A,B). No significant difference in superoxide dismutase (SOD) activity was observed between the control and drought stress conditions except in root SOD activity under low N concentration. However, high N concentration increased the SOD activities in the shoot and root by 12% and 22%, respectively; yet, the difference was not significant between drought and control conditions (Figure 6C,D). The root SOD activity under low N concentration significantly increased by 19% under drought stress as compared to the control condition (Figure 6D). In addition, high N concentration increased shoot peroxidase (POD) (32%), root POD (32%), and shoot catalase (CAT) (28%) activities compared with low N concentration (Figure 7A,B,D). These results suggest that the increase in antioxidant enzymatic activities were closely related to high N concentration, which can help in drought stress alleviation.

### 2.4. Nitrogen Use Efficiency Traits

**Figure 8:**
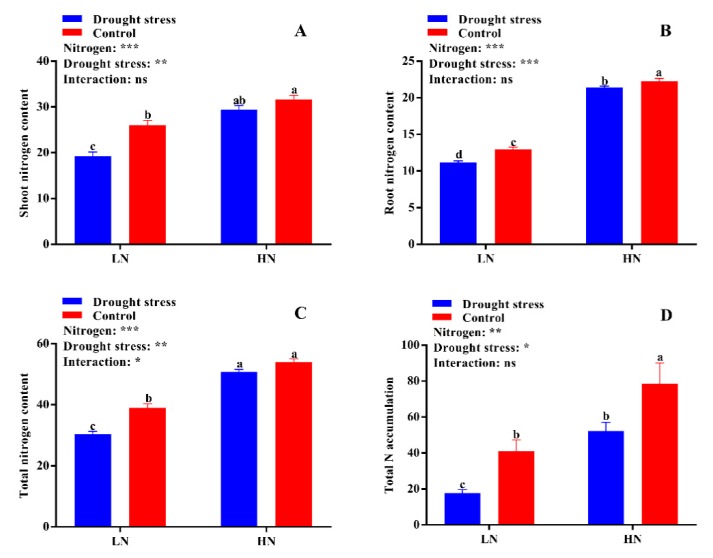
(**A**) Shoot nitrogen content (mg g^− 1^), (**B**) root nitrogen content (mg g^−1^), (**C**) total nitrogen content (mg g^−1^), and (**D**) total nitrogen accumulation (mg N) of cotton seedlings at the end of the experiment. Control: 0 g L^−1^ PEG-6000; drought stress: 150 g L^−1^ PEG-6000; LN, low N concentration (0.25 mM); HN, high N concentration (5 mM). Bars with different letters indicate significant difference (*p* < 0.05). Error bars represent the standard error (*n* = 3). *p*-Values of the ANOVA of nitrogen, drought, and their interaction are indicated as ns, not significant; * *p* < 0.05, ** *p* < 0.01, *** *p* < 0.001.

Nitrogen contents (shoot, root, and total) and accumulation were significantly affected by the N concentrations and drought stress. The results showed that drought stress significantly reduced shoot, root, and total N contents and total N accumulation under low N concentration (Figure 8). High N concentration significantly increased the shoot (26%) and total N contents (34%) irrespective of drought and control conditions (Figure 8A,C), but the root N content (8%) and total N accumulation (41%) were higher only under the control condition (Figure 8B,D).

**Figure 9:**
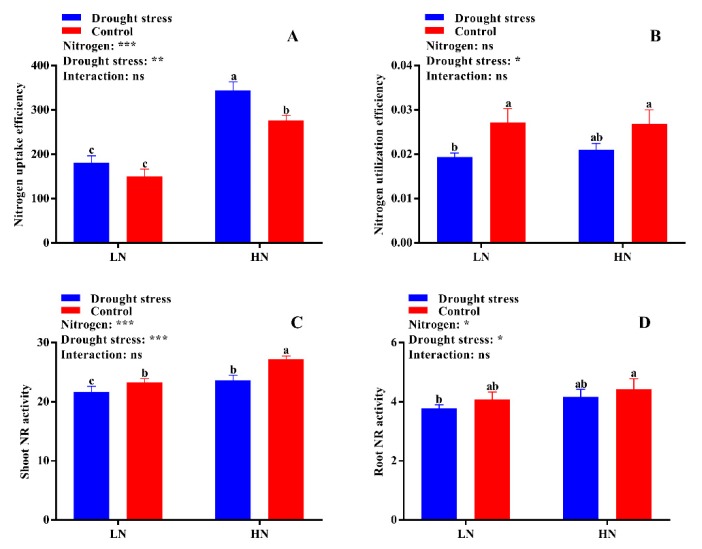
(**A**) Nitrogen uptake efficiency (mg N g^−1^ RDW), (**B**) nitrogen utilization efficiency (g^2^ DW mg^−1^ N), (**C**) shoot nitrogen reductase (NR) activity (µg g^−1^ FW h^−1^), and (**D**) root NR activity (µg g^−1^ FW h^−1^) of cotton seedlings at the end of the experiment. Control: 0 g L^−1^ PEG-6000; drought stress: 150 g L^−1^ PEG-6000; LN, low N concentration (0.25 mM); HN, high N concentration (5 mM). Bars with different letters indicate significant difference (*p* < 0.05). Error bars represent the standard error (*n* = 3). *p*-Values of the ANOVA of nitrogen, drought, and their interaction are indicated as ns, not significant; * *p* < 0.05, ** *p* < 0.01, *** *p* < 0.001.

N uptake efficiency was significantly reduced under low N concentration irrespective of drought and control conditions (Figure 9A). In contrast, high N concentration increased N uptake efficiency by 47% under drought stress as compared to the control (Figure 9A). Drought stress decreased N utilization efficiency by 25% under low N concentration (Figure 9B). However, high N concentration maintained the N utilization between control and drought stress conditions (Figure 9B). Thus, under drought stress conditions, high N concentration is a better source to maintain or increase N uptake and utilization efficiency.

### 2.5. Nitrogen-Assimilating Enzymatic Activities

**Figure 10:**
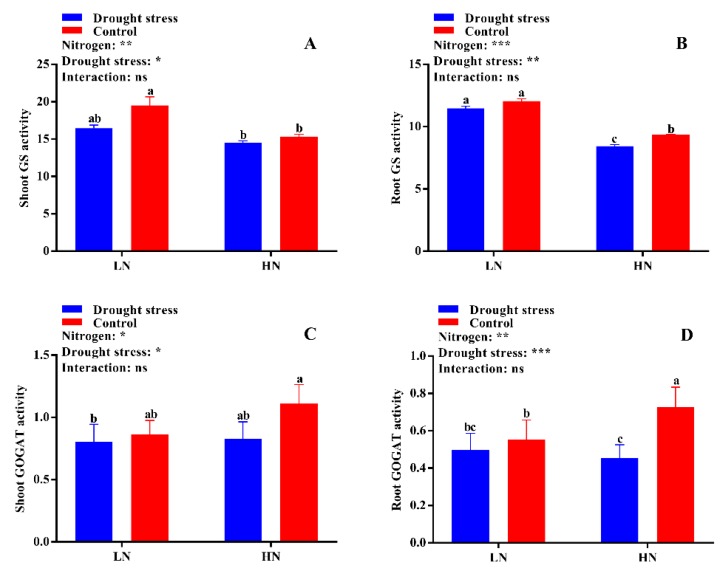
(**A**) Shoot glutamine synthetase (GS) activity (µmol g^−1^ FW h^−1^), (**B**) root GS activity (µmol g^−1^ FW h^−1^), (**C**) shoot glutamate synthase (GOGAT) activity (U mg^−1^ protein), and (**D**) root GOGAT activity (U mg^−1^ protein) of cotton seedlings at the end of the experiment. Control: 0 g L^−1^ PEG-6000; drought stress: 150 g L^−1^ PEG-6000; LN, low N concentration (0.25 mM); HN, high N concentration (5 mM). Bars with different letters indicate significant difference (*p* < 0.05). Error bars represent the standard error (*n* = 3). *p*-Values of the ANOVA of nitrogen, drought, and their interaction are indicated as ns, not significant; * *p* < 0.05, ** *p* < 0.01, *** *p* < 0.001.

**Figure 11:**
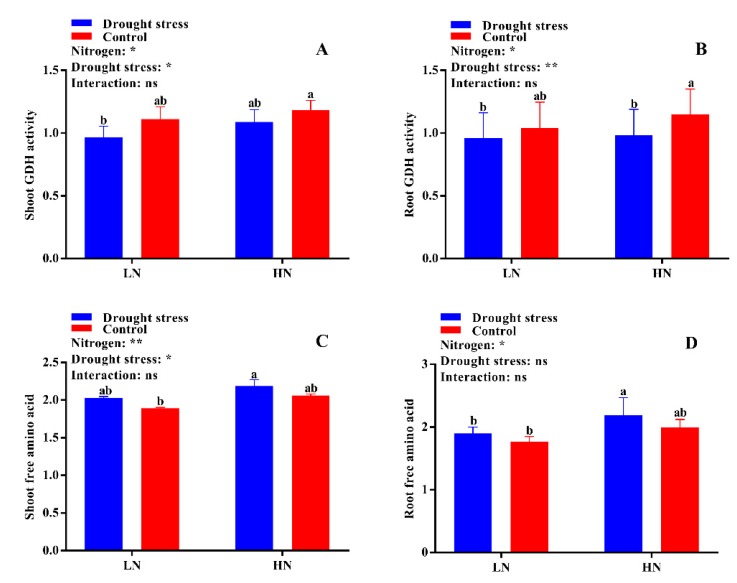
(**A**) Shoot glutamate dehydrogenase (GDH) activity (U mg^−1^ protein), (**B**) root GDH activity (U mg^−1^ protein), (**C**) shoot free amino acid (mg g^−1^ FW), and (**D**) root free amino acid (mg g^−1^ FW) of cotton seedlings at the end of the experiment. Control: 0 g L^−1^ PEG-6000; drought stress: 150 g L^−1^ PEG-6000; LN, low N concentration (0.25 mM); HN, high N concentration (5 mM). Bars with different letters indicate significant difference (*p* < 0.05). Error bars represent the standard error (*n* = 3). *p*-Values of the ANOVA of nitrogen, drought, and their interaction are indicated as ns, not significant; * *p* < 0.05, ** *p* < 0.01.

The activities of different N-assimilating enzymes were also measured in the current study to know the extent of N metabolism under drought stress in cotton seedlings. Drought stress significantly decreased shoot NR activity under both low and high N concentrations as compared to the control. However, no significant difference between drought and control was observed for root NR activity irrespective of N concentrations (Figure 9D). High N concentration significantly increased shoot NR activity (12%) in the control condition as compared to drought stress (Figure 9C). The difference between the control and drought stress for GS activity was not significant irrespective of N concentration (Figure 10A,B). However, higher shoot and root GS activities were observed under low N concentration compared with high N concentration. Specifically, root GS activity was significantly higher in high N concentration in the control as compared to drought (Figure 10B). The GOGAT activity was similar in control and drought stress conditions, except root GOGAT activity increased by 11% under high N concentration in the control (Figure 10C,D). Similarly, no significant difference was observed in tissue glutamate dehydrogenase (GDH) activity except for root GDH activity under high N concentration in the control, where root GDH activity was 6% higher than that under low N concentration (Figure 11A,B). In summary, the N-assimilating enzymatic activities were improved under high N concentration except GS. The differences between the drought and control were not significant for most of the tissue’s enzymatic activities, indicating that high N maintains the activities of N-assimilating enzymes by alleviating the negative impact of drought on N metabolism.

### 2.6. Free Amino Acid, Soluble Protein, and Total Soluble Sugar Contents

**Figure 12:**
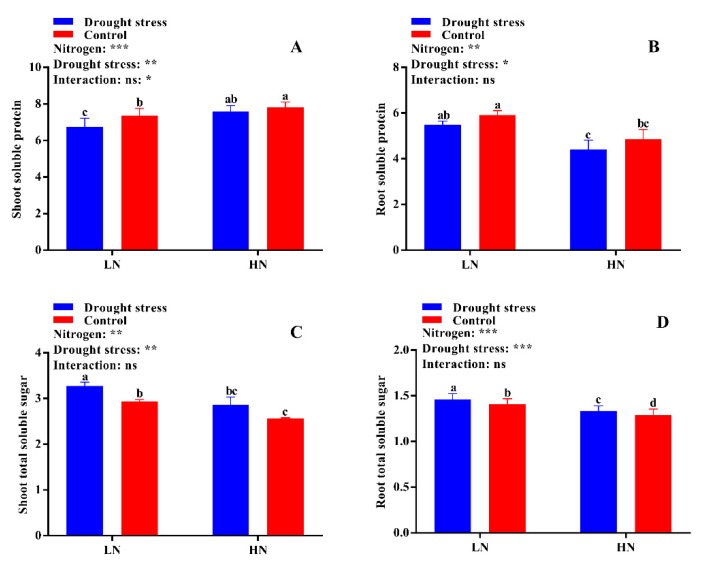
(**A**) Shoot soluble protein (mg g^−1^ FW), (**B**) root soluble protein (mg g^−1^ FW), (**C**) shoot soluble sugar (mg g^−1^ FW), and (**D**) root soluble sugar (mg g^−1^ FW) contents of cotton seedlings at the end of the experiment. Control: 0 g L^−1^ PEG-6000; drought stress: 150 g L^−1^ PEG-6000; LN, low N concentration (0.25 mM); HN, high N concentration (5 mM). Bars with different letters indicate significant difference (*p* < 0.05). Error bars represent the standard error (*n* = 3). *p*-Values of the ANOVA of nitrogen, drought, and their interaction are indicated as ns, not significant; * *p* < 0.05, ** *p* < 0.01, *** *p* < 0.001.

The differences between drought stress and control conditions were not significant for tissue free amino acid contents, however, a slight increase in both shoot and root free amino acid content was observed under high N concentration in drought conditions (Figure 11D). Consistently, drought stress decreased shoot soluble protein by 8% under low N concentration. Under high N concentration, no significant difference for shoot soluble protein was observed between drought stress and the control (Figure 12A). Similarly, no significant difference between drought stress and the control was observed for root soluble protein irrespective of the N concentrations (Figure 12B). Moreover, drought stress significantly increased shoot and root soluble sugar contents irrespective of the N concentrations (Figure 12A,B). However, the difference between drought and control was not significant for shoot soluble sugar contents (Figure 12C). In short, shoot soluble protein under drought stress was improved by high N concentration. However, the high soluble sugar contents under low N concentration might be due to translocation of more carbohydrates to roots under low N concentration as observed by the high root morphological traits.

### 2.7. Principal Component Analysis of Morphophysiological and Biochemical Traits

**Figure 13:**
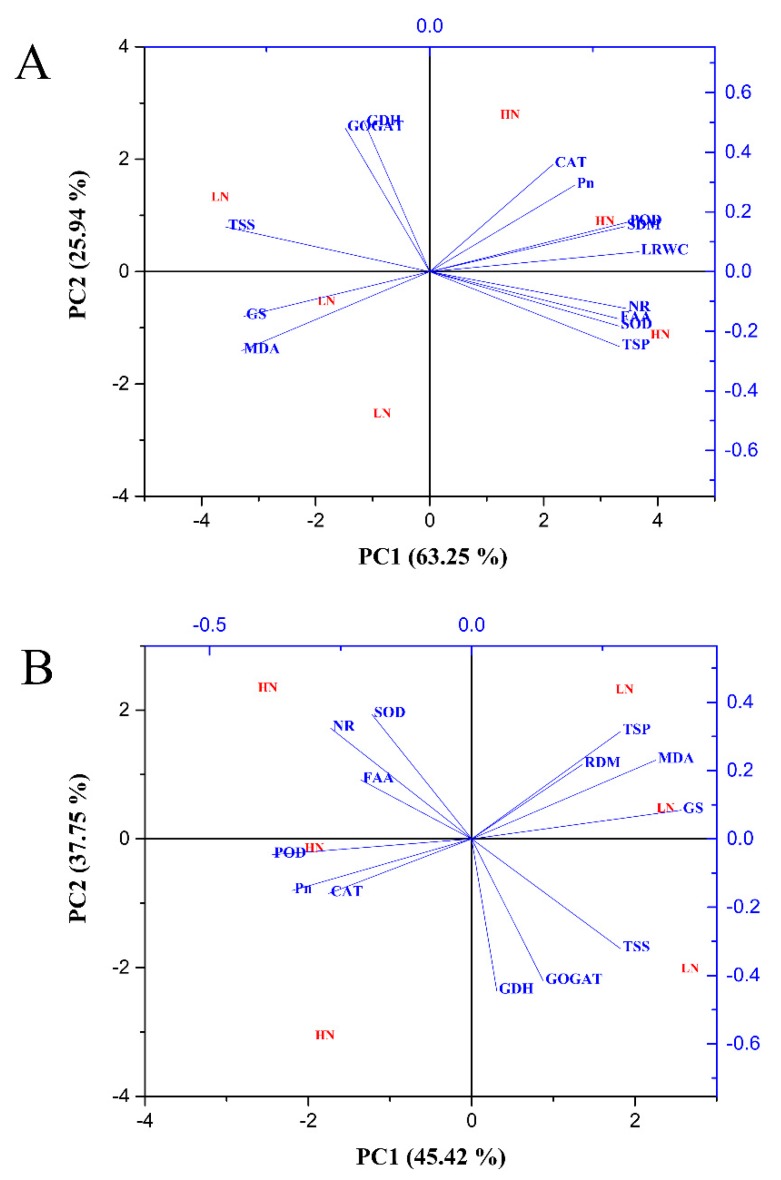
Principal component analysis (PCA) biplot of (**A**) shoot and (**B**) root morphophysiological and biochemical traits under combined N concentrations and drought stress conditions. The eigenvectors are shown in Table S1.

Principal component analysis was performed to determine the key traits involved in the response of N-treated cotton seedlings to drought stress conditions. In the shoot, PC1 and PC2 comprised 45.09% and 23.04% of the variation, respectively (Figure 13A). PC1 showed that the traits were differentially affected by drought stress treatment. PC2 showed that distinct variation resulted from the effect of N concentrations (Figure 13A). Total soluble protein, NR activity, shoot dry matter, POD activity, free amino acid content, SOD activity, and photosynthetic activity mainly contributed to PC1 (Table S1), whereas photosynthetic activity, GS activity, NR activity, shoot dry matter, and GOGAT activity were essential factors for PC2 (Table S1). For root traits, PC1 and PC2 represented 45.42% and 37.75% of the variation, respectively (Figure 13B). GS activity, MDA content, total soluble protein, total soluble sugar, and shoot dry matter were contributors to PC1, while SOD activity, NR activity, total soluble protein, MDA content, and shoot dry matter were the main factors for PC2 (Table S1). The PC1 values were higher than those of PC2, which suggests that drought stress had a large impact on the morphophysiological and biochemical traits of cotton. The greater distance between low and high N concentration indicated the sensitivity of low N-treated plants compared with high N-treated plants under drought stress (Figure 13).

## 3. Discussion

### 3.1. High Nitrogen Enhances Morphophysiological Tolerance of Cotton under Drought Stress

In the current study, different morphological changes were observed in cotton in response to PEG-induced drought stress under low and high N concentrations (Figure 1). Significantly reduced growth was observed under drought stress conditions. We assumed that the reduction in growth under drought might be due to a reduction in leaf physiological traits like chlorophyll contents, photosynthetic rate, and nitrogen assimilation, which result in poor carbohydrate production and low N metabolism. Consistent with current results, previous studies showed a significant reduction in growth and many physiological processes such as photosynthesis and related traits under drought stress [21,33]. The reduction in these physiological processes under drought stress resulted in many morphological changes such as decreased shoot length, leaf area, dry biomass, and root architecture, etc. [34]. This sensitivity of morphological traits and photosynthesis to drought stress could contribute to the different performances of biomass and photosynthetic rate in response to drought stress. The significant effect of N concentrations and drought stress interaction on photosynthetic rate implies that N is essential for regulating the adaptation of photosynthesis to drought stress. As a result, seedling’s growth increased due to the positive effects of high N concentration on stress alleviation. In line with the current results, better growth performance was found in *Populus simonii* with high N concentration under drought stress, suggesting that increasing N availability may help plants to survive drought stress conditions [34].

Drought stress tolerance of plants is significantly governed by its water retention capacity [35]. Transpiration is the major pathway of water loss from leaves. Generally, plants reduce water loss by partial closing of stomata under drought stress conditions. Consistent with this, stomatal conductance and transpiration rate significantly reduce, and as a result, leaf relative water content (LRWC) is maintained even under drought stress. It was suggested that higher water acquisition capacity under drought stress may contribute to a higher photosynthetic rate [36,37].

Additionally, studies confirmed that most N is used in the photosynthetic system [38] for regulating stomatal conductance and carbon dioxide diffusion [27]. Stomatal conductance is very important for photosynthesis, especially under drought stress conditions, as it regulates gas and water exchange between the leaf and external environment [39]. In the current study, drought stress and its interaction with N showed significant effects on stomatal conductance, and a stomatal limitation to photosynthesis occurred at low N concentration (Figure 4B), suggesting that high N is a pivotal factor in regulating stomata movement under water scarcity. Thus, high N concentration improves the sensitivity of stomata and alleviates stomatal limitation of photosynthesis in drought stress conditions [27,31]. A strong positive relationship between N and stomatal conductance (gs) was noted in previous studies [40,41]. Moreover, the increased resistance of photosynthesis to drought stress under high N concentration may also be due to an increase in antioxidant enzymatic activities [28] and nitrogenous compounds, especially proline, which helps plants maintain water balance under stress conditions [32]. However, further studies may help to elucidate the role of high N concentration in stomatal regulation and leaf photosynthesis under drought stress conditions.

### 3.2. High Nitrogen Improves Antioxidant Enzymatic Activities under Drought Stress

Studies have found that drought stress disturbs the equilibrium of reactive oxygen species (ROS) utilization and accumulation [42]. This increases photo-oxidative damage to photosynthesis and peroxidation of the cell membrane [43]. Therefore, for normal photosynthesis, the maintenance of ROS is very important to avoid photosynthetic damage in plants [44] and to maintain redox equilibrium inside the cell [45]. Plants have many tools to maintain ROS equilibrium, one of which is antioxidant systems like SOD, POD, and CAT [28]. Here, cotton seedlings show a good resistance system to alleviate the damage caused by oxidative stress. Significant increases in the activities of SOD, POD, and CAT were observed in cotton seedlings treated with high N concentration under drought stress conditions (Figure 6C,D and Figure 7). The enhanced antioxidant enzymatic activities and reduction in MDA content under high N concentration indicate an increase in the redox defense system in response to drought stress (Figure 6 and Figure 7). Increases in the activities of SOD, POD, and CAT, as well as soluble protein, were noted in maize leaves under high N concentration [46]. Drought stress regulates the activities of antioxidant enzymes in plants [35]. Moreover, the capacity of antioxidant enzymes greatly depends on N concentration. The application of high N concentration improves antioxidant enzymatic activities and thus reduces lipid peroxidation [47]. Similarly, high N concentration improved drought stress tolerance in rice by preventing cell membrane damage through antioxidant enzymatic activities [27]. In contrast, low nitrogen results in poor ROS scavenging capacity and thus increases oxidative stress [48]. In the current study, high lipid peroxidation and low antioxidant activities were observed under low N concentration (Figure 6 and Figure 7), which are consistent with previous results [27]. Thus, application of high N concentration might be a better strategy to enhance drought tolerance in cotton through high antioxidant enzymatic activities, which prevents cell membrane damage by scavenging ROS and promoting effective dissipation of energy.

### 3.3. High Nitrogen Balances Nitrogen Use Efficiency and Metabolism in Drought Stress

Nitrogen (N) and carbon metabolism are interlinked in the biochemical pathways of plants. N assimilation, like nitrate reduction, consumes energy [49] to form different amino acids and is dependent on the reaction site [44].

In the shoot, the reduction of nitrate consumes energy or excessive reducing power derived from the leaf photosynthetic process, and is comparatively more efficient than the reaction in the roots under drought stress conditions [50]. In our study, tissues’ N content was significantly increased under high N concentration (Figure 8A–C) and consequently improved both N uptake and utilization efficiency (Figure 9A,B). Previously, many studies confirmed the strong relationship between plant stress tolerance and N uptake and utilization [27,51,52,53]. However, it largely depends on stress intensity and N concentration [27]. In the current study, the better growth, N use efficiency, and metabolism under high N concentration are considered as key to tolerating drought stress, which is consistent with previous studies that high N concentration improves growth and N uptake under drought stress as compared to low N concentration [28,29,30].

The varying rates of N uptake by the root in response to drought stress and N concentration may lead to different N statuses and N-metabolizing enzymatic activities [34]. Thus, drought might alter N metabolism and allocation through the regulation of enzymes involved in N assimilation. In the current study, the enzymes were more sensitive under low N concentration and drought stress greatly affected the activities of NR, GS, GOGAT, and GDH, which are consistent with the findings of Wang et al. [54] who observed that drought stress alters N uptake and assimilation. Drought decreases growth due to the high sensitivity to leaf area expansion to plant water status; therefore, drought stress also affects N demand [50]. Leaf area can act as an indicator of N demand [43]. In the current study, we found that leaf area and LRWC were decreased under drought stress, indicating that water limitation leads to a reduced shoot N demand, and thus a decrease in N-assimilating enzymatic activities. In line with our results, it has been widely reported that the activities of key enzymes related to N assimilation were consistently down-regulated under drought stress [19,55,56]. However, our results displayed that the response of N-assimilating enzymes to drought stress was mainly dependent on N concentrations. These enzymes were more sensitive to drought stress under low N concentration, and drought stress significantly reduced the activities of N-assimilating enzymes. The low N assimilation in the current study is consistent with previous results, confirming that drought stress significantly reduced the activities of N-assimilating enzymes [19,34,55,56]. Simultaneously, high N concentration increased N-assimilating enzymatic activities, suggesting that high N concentration mitigates the negative effects of drought stress on N metabolism.

Plants rely on osmotic regulation to overcome stress, and their osmotic potential reflects their capacity for stress tolerance [57]. PEG-induced drought stress also alters the levels of nitrogenous compounds like free amino acids, total soluble proteins, and sugars in cotton seedlings, which help in osmotic regulation during drought stress conditions [58]. Cotton seedlings maintain osmoregulation by changing the levels of these osmoprotectants under drought stress conditions, which is considered as osmotic stress tolerance [59]. The variations in free amino acids under drought stress at low and high N concentrations reflect the accumulation of free amino acids [60], which acts as a stress indicator [22]. Amino acids are well-known osmotic regulators that help to maintain cell turgidity and avoid tissue dehydration [61]. In the current study, the increased free amino acids serve as osmotic regulators and contribute to reducing osmotic potential and thus maintain cell turgidity, as shown by high LRWC. Therefore, this study suggests that high N concentration can maintain high N metabolism and levels of osmoprotectants and hence increase resistance to drought stress. Moreover, soluble proteins and sugars are the main cellular components that act as catalysts and improve tolerance in plants under stress conditions [59,62]. Many scientists have studied and confirmed the production and accumulation of osmoprotectants under stress conditions in different plants [63,64,65]. We also concluded that cotton seedlings treated with high N concentration under drought stress conditions increase the production and accumulation of osmoprotectants like free amino acids and total soluble proteins, which help in maintaining osmotic potential and cell turgidity, thus avoiding dehydration of the plant tissues.

## 4. Materials and Methods

### 4.1. Plant Growth Conditions and Treatments

A hydroponic experiment was carried out in a growth chamber at the Institute of Cotton Research of the Chinese Academy of Agricultural Science, Anyang, China. Seeds of the cotton cultivar “TM-1” were germinated in a mixture of sand and vermiculite for one week in a germinator. After the full opening of two cotyledons, healthy and uniform seedlings were transplanted into 7 L plastic containers in a growth chamber (16/8 h light/dark cycle, 28 °C light/dark temperature regime, 60% relative humidity). During the first week, half-strength Hoagland solution was used, followed by full-strength solutions (1 mM KH_2_PO_4_, 2 mM KCL, 2 mM MgSO_4_, 0.1 mM EDTA·Fe·Na, 46.2 uM H_3_BO_3_, 9.1 uM MnCl_2_·4H_2_O, 0.8 uM ZnSO_4_·7H_2_O, 0.3 uM CuSO_4_ 5H_2_O, 1.0 uM (NH_4_)6Mo_7_O_24_ 4H_2_O) till end of the experiment. At three leaves stage, seedlings were separated into four experimental groups: (1) low N concentration without drought stress (0.25 mM+ control); (2) low N concentration with drought stress (0.25 mM + 150 g PEG L^−1^); (3) high N concentration without drought stress (5 mM+ control); and (4) high N concentration with drought stress (5 mM + 150 g PEG L^−1^). In low N concentration, 1 mM L^−1^ CaCl_2_ was also supplied to compensate calcium in the medium [54]. The nutrient solution was refreshed every week and aerated using an electric pump. The position of pots was interchanged when refreshing the solution to eliminate the edge effects.

### 4.2. Plant Growth and Root Morphology

Plant growth was measured by using six uniform plants from each replication. Shoot length of four randomly selected plants was measured with a ruler in each replication. The average of all the plants from treatments was worked out for mean shoot length. Similarly, lengths and widths of each leaf of six randomly selected plants were measured, then the mean single leaf area was calculated [66]. At the end of the experiment, seedlings were harvested and separated into shoot and roots, placed in labeled paper bags. The samples were dried in an electric oven at 105 °C for half an hour followed by 80 °C for 72 h. Once completely dried, the dry weight of shoot and root was measured on an electric balance. At the end of the experiment, the root of six plants from each treatment was excised and scanned through WinRHIZO root analyzer system (WinRHIZO version 2012b, Regent Instruments Canada, Montreal, QC, Canada) for various root morphological traits.

### 4.3. Gas-Exchange Measurements and LRWC

At the end of a drought stress episode, photosynthetic measurement was conducted on the youngest fully expanded leaves (second from top) randomly selected from six plants with the help of a photosynthetic machine (Li-Cor-6800; Li-Cor, Inc., Lincoln, NE, USA) under 1500 µmol m^2^ s^2^ light intensity, 32 °C leaf temperature, and 380 µmol mol^−1^ CO_2_ concentrations from 9:00 to 11:00 a.m. in the growth chamber. The relative chlorophyll content was measured with a portable chlorophyll meter (SPAD 502 Meter, Minolta Corporation, Tokyo, Japan). Leaf relative water content (LRWC) was estimated as LRWC (%) ((Fw − Dw)/(Sw − Dw)) × 100. Water-saturated weight (Sw) of 0.4 g fresh weight (Fw) leaf samples were obtained by keeping leaf disks in distilled water for 6 h. Then the samples were oven-dried at 70 °C to get a constant dry weight (Dw) [67].

### 4.4. Determination of Chlorophyll and Carotenoid Content

For the determination of leaf chlorophyll and carotenoid contents about 50 mg of fresh samples were collected. The leaves were cut into small pieces and incubated in a mixture of acetone and anhydrous ethanol solution (1:1) under the dark condition at 25 °C for 48 h. The absorbance for chlorophyll and carotenoid contents was measured according to the protocol developed by [68].

### 4.5. Measurement of N Concentration, N Accumulation, and N Use Efficiency Traits

Nitrogen in the plant tissues was measured through the Kjeldahl method. The shoot and root dried samples were ground and around 0.2 g of each sample powder was weighed, digested with H_2_SO_4_-H_2_O_2_, and then analyzed for N content using the Bran + Luebbe Continuous-Flow AutoAnalyzer III (AA3). Different N use efficiency (NUE) definitions were estimated from the values of N concentrations [69]. The total N accumulation was obtained as the product of N concentrations and plant total dry weight [70]. N utilization efficiency was measured as total plant dry weight divided by N concentrations [71] and N uptake efficiency was determined as the ratio of N accumulation and root dry weight [72].

### 4.6. Determination of Lipid Peroxidation and Antioxidant Enzymatic Activities

To determine lipid peroxidation, 0.5 g fresh samples were used and the data were obtained using the published protocol [73]. Similarly, 0.5 g fresh sample was taken to assess antioxidant enzymatic activities in 5 mL of 50 mM phosphate buffer solution. The superoxide dismutase (SOD; EC 1.15.1.1), peroxidase (POD; EC 1.1.1.1.7), and catalase (CAT; EC 1.11.1.6) activities were measured [74,75,76].

### 4.7. Measurement of N-Assimilating Enzymatic Activities

The activities of nitrate reductase (NR; EC 1.7.1.3) and glutamine synthetase (GS; EC 6.3.1.2) were determined according to Silveira et al. [77] and Husted et al. [78], respectively. Glutamate synthase (GOGAT; EC 1.4.7.1) activity was measured by taking extraction buffer containing 10 mmol L^−1^ Tris HCL (pH 7.6), 1 mmol L^–1^ MgCl_2_, 1 mmol L^–1^ Ethylene diamine tetra acetic acid (EDTA), and 1 mmol L^–1^ mercaptoethanol. About 0.5 g of the fresh root and shoot samples were ground in liquid nitrogen followed by centrifugation at 12,000× *g* at 4 °C for 25 min. The enzymatic activity was measured by absorbance 340 nm for 3–4 min at room temperature [79]. For determination of glutamate dehydrogenase (GDH; EC 1.4.1.2) activity, the previously published method was followed [80].

### 4.8. Measurement of Total Soluble Protein, Total Free Amino Acids, and Sugar

Total soluble protein was measured according to the method used by Bradford [81], using albumin bovine [82]. About 0.5 g root and shoot samples were homogenized in phosphate buffer (5 mL). The samples were placed in a water bath 100 °C for 10 min and centrifuged at 3000× *g* for 5 min at 22–25 °C. The reaction mixture was composed of 2 mL d H_2_O, enzyme extract (20 µL), and Bradford reagent (0.5 mL). Finally, the values were recorded at 595 nm wavelength using distilled water as a blank control with the help of a spectrophotometer (UV-2600).

The total free amino acid was measured by the previously used method [83] with some modifications [84]. Extraction buffer composed of acetic acid/sodium acetate (pH 5.4) and the final values of free amino acids were detected at 580 nm using a spectrophotometer (UV-2600).

Total soluble sugar was determined as suggested by Shields and Burnett [85] followed with some modifications. The shoots and roots samples (0.5 g) were ground into fine powder in liquid nitrogen using a mortar and pestle. The samples were homogenized in 90% ethanol (3 mL) and then put at 60–70 °C for incubation. After incubation, 90% ethanol was again added to the extract in a volumetric flask (final vol. 25 mL). Each sample (1 mL) was then mixed with anthrone solution (5 mL) and sulfuric acid (5 mL). The final value was then observed at 485 nm using glucose as standard.

### 4.9. Statistical Analysis

Two-way ANOVA was performed to analyze the impacts of nitrogen and drought stress on cotton seedlings using Statistix 10 (Analytical Software, Tallahassee, FL, USA). A least significant difference (LSD) was performed for mean comparison at 5% level of significance. The Graphpad Prism 7.0 software (GraphPad Software, La Jolla California USA, www.graphpad.com) was used for making figures, while principal component analysis (PCA) was performed through OriginPro 2016 (Origin Lab Corporation, Northampton, MA, USA). The data are expressed as the mean ± standard error (SE) of triplicates.

## 5. Conclusions

**Figure 14:**
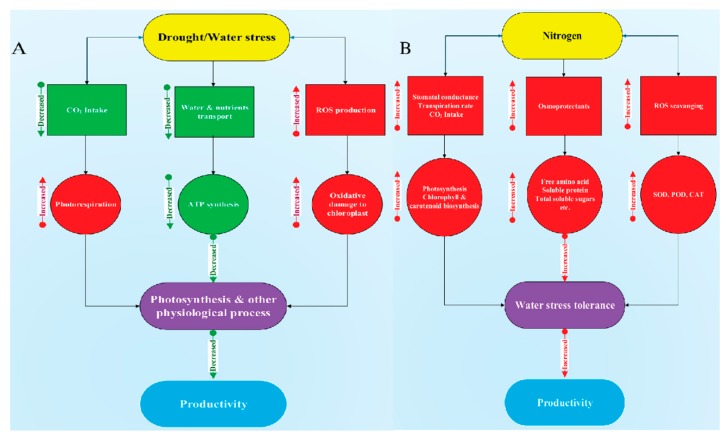
Diagrammatic representation of drought stress impacts on (**A**) cotton and (**B**) its alleviation through high N concentration.

In summary, PEG-induced drought stress limited cotton growth, photosynthetic activity, N uptake, and assimilation. However, high N concentration maintained a high photosynthetic rate, nitrogen uptake, and utilization to ensure normal growth under drought stress conditions. Moreover, high N concentration mitigates stomatal limitation, maintains leaf relative water content, increases the activities of antioxidant enzymes (SOD, POD, and CAT), N-assimilating enzymes (NR, GS, GOGAT, and GDH), and synthesis of osmoprotectants (free amino acid and soluble protein). The results suggest that cotton seedlings supplied with relatively higher N concentration may be beneficial to enhance drought stress tolerance in cotton (Figure 14). Moreover, the accumulation of free amino acids and reduction of N-assimilating enzymes in cotton under drought stress need to be studied at the molecular level to know the actual mechanisms of osmotic adjustment.

## Supplementary Materials

The following are available online at https://www.mdpi.com/2223-7747/9/2/178/s1, Figure S1: Root-shoot ratio (A), single leaf area (cm) (B), SPAD value (C) and root surface area (cm^2^) (D) of cotton seedlings at the end of the experiment. Control: 0 g L^−1^ PEG-6000, drought stress: 150 g L^−1^ PEG-6000, LN, low nitrogen concentration (0.25 mM) and HN, high nitrogen concentration (5 mM). Bars with the different letters indicate significant difference (*p* < 0.05). Error bars represent the standard error (n = 3). P-values of the ANOVAs of nitrogen, drought, and their interaction are indicated as ns; not significant, *; *p* < 0.05, **; *p* < 0.01.Figure S2 Root length ratio (%) (A), root mass ratio (cm) (B), root thickness (mm) (C) and root density (cm3) (D) of cotton seedlings at the end of the experiment. LN, low nitrogen (0.25 mM); HN, high nitrogen (5 mM). Control: 0 g L^−1^ PEG-6000, drought stress: 150 g L^−1^ PEG-6000, LN, low nitrogen concentration (0.25 mM) and HN, high nitrogen concentration (5 mM). Bars with the different letters indicate significant difference (*p* < 0.05). Error bars represent the standard error (n = 3). P-values of the ANOVAs of nitrogen, drought, and their interaction are indicated as ns; not significant, *; *p* < 0.05, **; *p* < 0.01. Table S1: PCA of morphophysiological and biochemical traits of shoot and root under combined conditions of nitrogen and water stress.
