# Supplementary material for: High Nitrogen Enhance Drought Tolerance in Cotton through Antioxidant Enzymatic Activities, Nitrogen Metabolism and Osmotic Adjustment
Source: Plants (Basel). 2020 Feb 1;9(2):178. doi: 10.3390/plants9020178 (PMC7076502; doi:10.3390/plants9020178)
Supplement: Supplementary file 1 [file plants-09-00178-s001.pdf]

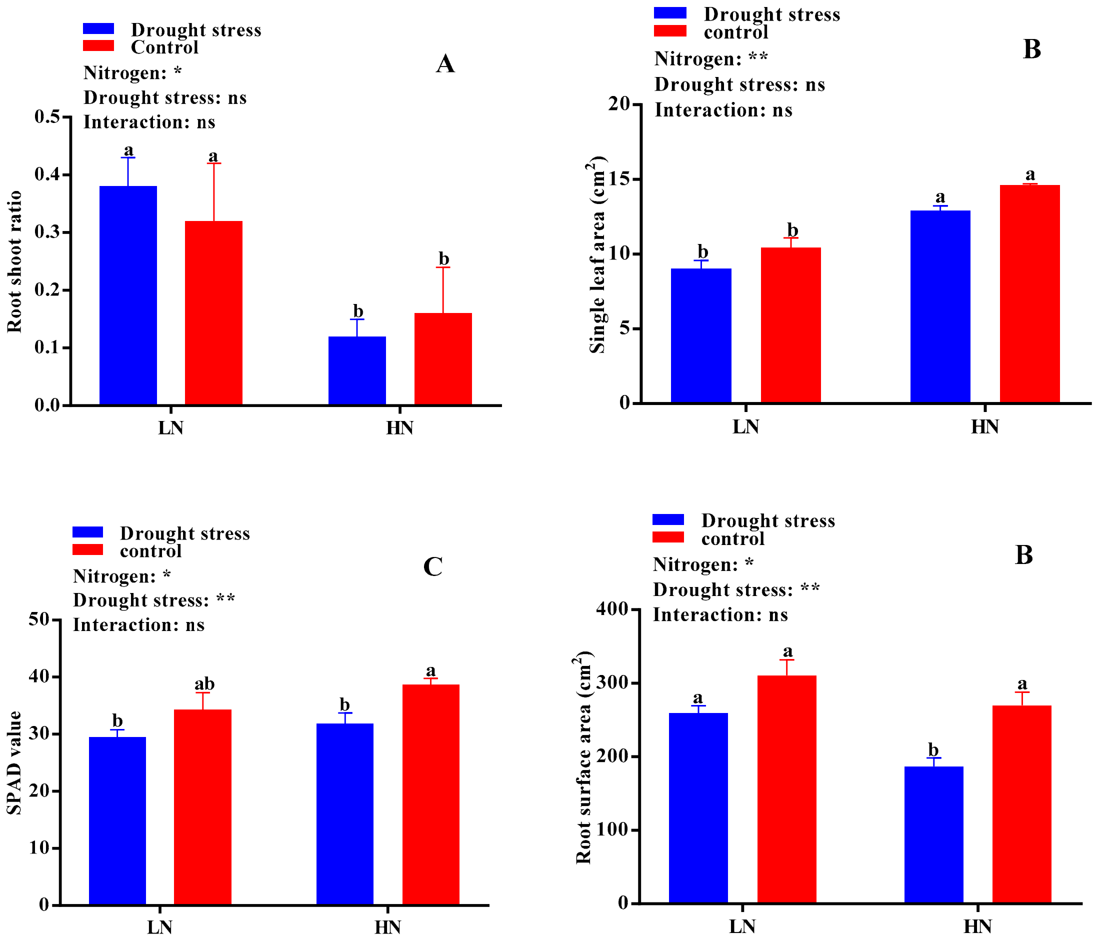


Supplementary figure 1. Root-shoot ratio (A), single leaf area (cm) (B), SPAD value (C) and root surface area ($cm^2$) (D) of cotton seedlings at the end of the experiment. Control: 0 g $L^{-1}$ PEG-6000, drought stress: 150 g $L^{-1}$ PEG-6000, LN, low nitrogen concentration (0.25 mM) and HN, high nitrogen concentration (5 mM). Bars with the different letters indicate significant difference ($p < 0.05$). Error bars represent the standard error ($n = 3$). p-Values of the ANOVA of nitrogen, drought, and their interaction are indicated as ns, not significant; * $p < 0.05$, ** $p < 0.01$.

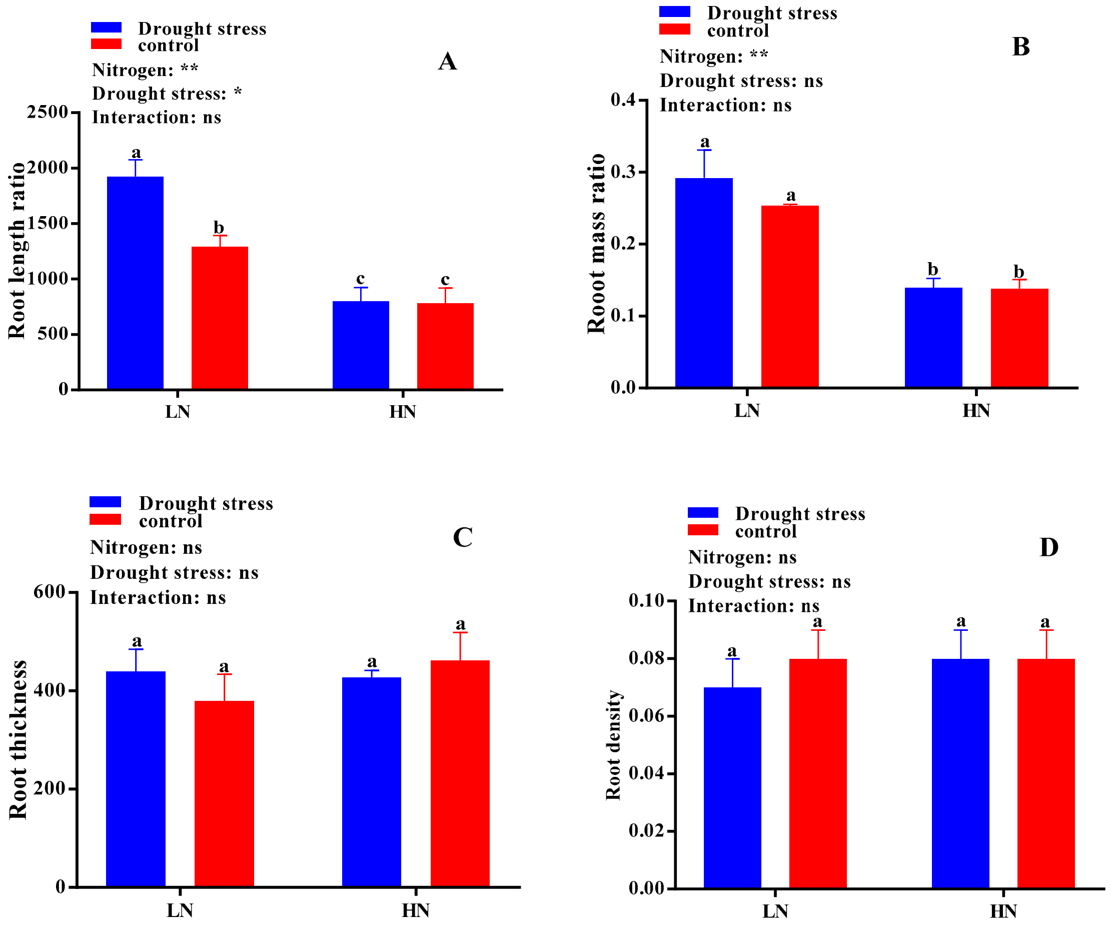


Supplementary figure 2. Root length ratio (%) (A), root mass ratio (cm) (B), root thickness (mm) (C) and root density ($cm^3$) (D) of cotton seedlings at the end of the experiment. LN, low nitrogen (0.25 mM); HN, high nitrogen (5 mM). Control: 0 g $L^{-1}$ PEG-6000, drought stress: 150 g $L^{-1}$ PEG-6000, LN, low nitrogen concentration (0.25 mM) and HN, high nitrogen concentration (5 mM). Bars with the different letters indicate significant difference ($p < 0.05$). Error bars represent the standard error ($n = 3$). P-Values of the ANOVAs of nitrogen, drought, and their interaction are indicated as ns; not significant, *; $p < 0.05$, **; $p < 0.01$.

Table S1. PCA of morphophysiological and biochemical traits of shoot and root under combined conditions of nitrogen and water stress.

| | Shoot | | Root | |
|---|---|---|---|---|
| | PC1 | PC2 | PC1 | PC2 |
| Total soluble sugar | -0.35 | -0.23 | **0.28** | -0.32 |
| Total soluble protein | **0.35** | 0.08 | **0.28** | **0.31** |
| Free amino acid | **0.28** | -0.35 | -0.21 | 0.17 |
| Nitrate reductase activity | **0.34** | **0.26** | -0.27 | **0.32** |
| Glutamate dehydrogenase activity | -0.17 | 0.14 | 0.05 | -0.45 |
| Glutamate synthase activity | -0.12 | **0.20** | 0.14 | -0.42 |
| Glutamine synthetase activity | -0.27 | **0.32** | **0.40** | 0.08 |
| Catalase activity | 0.10 | -0.24 | -0.27 | -0.16 |
| Peroxidase activity | **0.29** | -0.25 | -0.38 | -0.05 |
| Superoxide dismutase activity | **0.25** | -0.41 | -0.19 | **0.36** |
| Malondialdehyde content | -0.36 | -0.17 | **0.35** | **0.23** |
| Photosynthetic rate | **0.20** | **0.45** | -0.34 | -0.15 |
| Shoot dry matter | **0.34** | **0.26** | **0.21** | **0.22** |
| Eigen value | 5.86 | 2.99 | 5.90 | 4.90 |
| Variance contribution rate | 63.25 | 25.94 | 45.42 | 37.75 |
| Cumulative percentage | 45.09 | 68.13 | 45.42 | 83.17 |

The numbers in bold fonts indicate key factors of PC1 and PC2 with abstract of scores larger than 0.20.
